# Correlation of Maternal Stress Because of Positive Aneuploidy Screening Serum Analytes and Uterine Arteries’ Doppler Ultrasound Index: A Prospective Cohort Study

**DOI:** 10.22074/ijfs.2019.5359

**Published:** 2018-10-02

**Authors:** Mahboobeh Shirazi, Parichehr Pooransari, Fatemeh Rahimi Sharbaf, Shirin Niromanesh, Behrokh Sahebdel, Mamak Shariat, Zeinab Pahlavan, Mahmoud Shirazi, Maryam Ahmadian

**Affiliations:** 1 Maternal, Fetal and Neonatal Research Centre, Tehran University of Medical Sciences, Tehran, Iran; 2 Shohada Hospital, Shahid Beheshti University of Medical Sciences, Tehran, Iran; 3 Department of Perinatology, Ayatollah Rouhani Hospital, Babol University of Medical Sciences, Babol, Iran; 4 Department of Psychology, University of Sistan and Baluchestan, Zahedan, Iran

**Keywords:** Amniocentesis, Doppler, Prenatal Screening, Stress Disorders, Uterine Artery

## Abstract

**Background:**

Antenatal anxiety or maternal stress is a prevalent chronic mental disorder in pregnant women. We have assessed the effect of maternal stress from positive aneuploidy screening results on the changes in uterine artery blood flow.

**Materials and Methods:**

We performed a prospective cohort (one sample) pilot study at a hospital in Tehran, Iran. A total of 60 pregnant women who were candidates for amniocentesis due to abnormal sequential screening test results entered the study. We conducted 2 standard psychological tests, the Spielberger’s State-Trait Anxiety Inventory and the Beck Anxiety Inventory, to determine anxiety levels in the participants before amniocentesis and two weeks after amniocentesis. The uterine artery resistance index was also measured before and two weeks after amniocentesis. The level of maternal stress was compared with the uterine artery resistance index.

**Results:**

Patients had a mean State Trait Anxiety Inventory score before amniocentesis of greater than 40, which meant that the mothers experienced high anxiety. There were no correlations between both inventories’ anxiety scores and uterine artery blood flow before amniocentesis. However, two weeks after amniocentesis, we observed significant negative correlations between the State Anxiety (P=0.0041) and Trait Anxiety (P=0.010) Inventory scores and the uterine artery resistance indexes. Also, there was an association between the decreased right uterine artery resistance index and State Anxiety scores (P=0.036). There were significant correlations between State and Trait Anxiety scores and second trimester analytes of β-human chorionic gonadotropin (β-hCG, P<0.001), α-fetoprotein (P<0.001), and unconjugated estriol (P=0.048).

**Conclusion:**

Maternal anxiety because of positive aneuploidy screening serum analytes and amniocentesis can af- fect perinatal outcomes via mood-based alterations in blood flow of the uterine arteries and the screening markers β-hCG,unconjugated estriol, and α-fetoprotein.

## Introduction

Antenatal anxiety or maternal stress is a frequent complication in pregnant women (1). The prevalence of depression during pregnancy is 7-18% and anxiety disorders during pregnancy is 8.5% (2, 3). Mothers face tremendous anxiety because of pregnancy complications, labour pain, prenatal invasive procedures, the childbirth process, and concerns about the health and future of their infants (4).

Antenatal psychological disorders affect neonatal outcome and may increase the risk of spontaneous preterm delivery, low birth weight, caesarean section and instrumental delivery, as well as neonatal intensive care unit (NICU) admission (5, 6). Blood flow changes in the maternal foetal blood circulation, sympathetic arousal, and hypothalamicpituitary- adrenal axis activation are possible mechanisms behind adverse pregnancy outcomes (7, 8). Although determining the relationship between anxiety status and changes in maternal-foetal blood circulation is not easy (9), Doppler ultrasound measurements have demonstrated a correlation between uterine-umbilical blood circulation and maternal psychological disorders (10).

The sequential screening test is an aneuploidy screening test that measures β-human chorionic gonadotropin (β-hCG), pregnancy associated plasma protein A, α-fetoprotein, unconjugated estriol, and inhibin A (11-13), And is performed during the first trimester (10 weeks and 3 days until 13 weeks and 6 days) and second trimester (15-18 weeks). Several investigations have found a correlation between maternal anxiety and changes in these serum analytes (14-16). A few studies suggested an association between uterine blood circulation and maternal serum markers; pregnant women with increased β-hCG had abnormal Doppler ultrasound findings in uterine arteries from the 22^nd^ to 24^th^ pregnancy weeks (17).

Amniocentesis is an invasive diagnostic technique that rules out foetal aneuploidy. Studies have revealed that pregnant women experience high levels of anxiety as a result of positive aneuploidy screening tests, the amniocentesis procedure, uncertainty, tension, and foetal injury (18-20). A study has demonstrated high levels of long-lasting anxiety related to prenatal diagnostic procedures that include positive biochemical screening or ultrasound chromosomal abnormality markers. Some measures are suggested to decrease anxiety, and include promotion of the transparency of delivered information, as well as psychological and emotional interventions for both the medical team and high risk pregnant women (21).

This study investigated the correlation between anxiety scores related to positive aneuploidy screening serum analytes and uterine artery resistance index. To the best of our knowledge, few studies have focused on this subject. We assessed the correlation between screening serum analytes and psychological anxiety scales as the secondary outcome.

## Materials and Methods

This prospective cohort (one sample) pilot study was performed at Moheb Yas Hospital in Tehran in 2015. The study population consisted of 100 pregnant women who were candidates for amniocentesis because of positive sequential screening test results for aneuploidy (as exposure).

The inclusion criteria were: 20-37 years old, gestational age 15-20 weeks, 19-25 kg/m^2^ body mass index, high risk for sequential markers, and maternal mental health.

The exclusion criteria were history of psychological, psychotic, or neurocognitive disorders; smoking or drug abuse; underlying diseases such as hypertension, diabetes, recent experience of stressful events; and positive aneuploidy culture results after amniocentesis.

All participants signed an informed consent and accepted well-timed attending for receiving prenatal care. We used 2 standard psychological tests, the Spielberger’s State-Trait Anxiety Inventory (an index of obvious and hidden anxieties) and Beck’s Anxiety Inventory before amniocentesis and two weeks later to determine the participants’ anxiety level as the outcome.

All questionnaires were standard and their validities in the Iranian population were identified to be more than 91%. The State-Trait Anxiety Inventory is a 40-item self-report inventory in which scores more than 43 suggest high anxiety and scores that range from 15 to 20 indicate no anxiety. Beck’s Anxiety Inventory evaluates the responses to 21 multiple-choice, self-report questions to measure the severity of anxiety; a score of 16 or higher is considered moderate and severe anxiety (22, 23).

A single observer measured the uterine artery resistance index before amniocentesis using a Doppler ultrasound (ACUSON Sequoia 512™, Siemens Healthcare GmbH, USA) with a convex multi-frequency transducer (3.0 to 5.0 MHz). Patients were placed in the trans-abdominal position. After taking the images, the Doppler measurement was performed in location of the uterine artery that originates from the iliac artery just lateral to the cervix at the endocervix. We took into consideration an insonation angle of less than 30 degrees and a Doppler gate of 2 mm.

The same perinatologist performed another Doppler ultrasound two weeks after amniocentesis, after the culture results were available. The anxiety scores of all participants were recorded. Data related to serum analytes that included β-hCG and pregnancy associated plasma protein A in the first trimester, and α-fetoprotein, β-hCG, unconjugated estriol, and inhibin A in the second trimester were also recorded in the checklists (code and master sheets). Finally, we compared the effect of maternal stress based on high risk aneuploidy with the uterine artery resistance indexes before and two weeks after amniocentesis. The association between serum analytes and participants’ psychological status was also evaluated.

### Data analysis

The Statistical Package for Social Sciences (SPSS) software version 19 (Chicago, IL, USA) was used for statistical analysis. Questionnaires’ anxiety scores and variables related screening marker analytes and uterine artery resistance index were presented as mean standard deviation. In cases where the variables were normally distributed, we applied the paired t test and linear regression analysis with determination of beta coefficient and 95% confidence interval, to analyse the correlation between variables. This study had an 80% power. P<0.05 was considered significant.

This study was an experimental pilot study; therefore, we could not calculate the sample size. A total of 60 subjects entered the study from April to September 2015 based on the inclusion criteria and capability of the study design.

### Ethical considerations

The collected data was considered confidential and no extra costs were imposed on the participants. The participants received assurances about their right to withdraw from the study at any time. The Institutional Review Board of Tehran University of Medical Sciences, by taking into consideration the tenets of the Declaration of Helsinki, approved this study.

## Results

From among 100 pregnant women who met the inclusion criteria, 60 women entered the study and 40 women were excluded because of lack of cooperation or positive aneuploidy culture results. No participant required any medical treatment and all just received routine prenatal care. Participant had a mean age of 32 ± 3.52 years and their mean gestational age at the time of amniocentesis was 16 ± 0.56 weeks.

Pearson correlation test of the results showed a significant correlation between Beck’s Anxiety Inventory score with the State and Trait Anxiety Inventory scores (P<0.0001, r=0.570 and r=0.533).There was a significant association between the State and Trait Anxiety Inventory scores (P<0.0001, r=0.809)

The mean State Trait Anxiety Inventory scores before amniocentesis were higher than 40 (43.45 ± 7.86 and 42.23 ± 7.98, respectively), which indicated that mothers experienced high levels of anxiety. Although these mean values decreased after amniocentesis, there were no significant differences before and 2 weeks after amniocentesis (Table 1).

**Table 1:** Descriptive statistics of independent and dependent variables


Variables	Mean ± SD	Min	Max

Before amniocentesis			
Beck’s Anxiety Inventory	10.01 ± 8.99	1.00	43.00
Trait Anxiety Inventory	42.23 ± 7.98	26	66
State Anxiety Inventory	43.45 ± 7.86	30	66
Two weeks after amniocentesis			
Beck’s Anxiety Inventory	9.23 ± 7.44	2.00	40
Trait Anxiety Inventory	41.18 ± 8.44	25	62
State Anxiety Inventory	33.38 ± 7.66	24	52
Before amniocentesis			
Left artery resistance index	0.64 ± 0.15	0.33	0.93
Right artery resistance index	0.69 ± 0.15	0.34	0.92
Two weeks after amniocentesis			
Left artery resistance index	0.65 ± 0.17	0.27	1.02
Right artery resistance index	0.66 ± 0.18	0.16	0.94
Index changes			
Left artery resistance index change	0.01 ± 0.21	-60.59	0.57
Right artery resistance index change	-0.02 ± 0.22	-0.48	0.44
First trimester serum analytes			
Pregnancy associated plasma protein A	0.90 ± 0.49	0.03	1.80
β-human chorionic gonadotropin (β-hCG)	1.81 ± 1.30	0.01	4.6
Second trimester serum analytes			
β-hCG	2.14 ± 1.32	0.20	6.27
Inhib A	1.66 ± 0.80	0.34	3.56
α-fetoprotein	1.35 ± 1.50	0.23	7.28
Unconjugated estriol	0.67 ± 0.26	0.34	1.31


Min; Minimum and Max; Maximum.

Before amniocentesis, the left artery mean resistance index was 0.64 ± 0.15 and the right artery mean resistance index was 0.69 ± 0.15. At 2 weeks after amniocentesis, the mean resistance index for the left artery was 0.65 ± 0.17 and it was 0.66 ± 0.18 for the right artery. Table 2 shows that no significant association existed before amniocentesis between Beck’s Anxiety Inventory scores and the left uterine artery resistance index (P=0.394, β=0.141, 95% CI:-0.003 to 0.008) as well as the right uterine artery resistance index (P=0.897, β=0.021, 95% CI:-0.005 to 0.006). At 2 weeks after amniocentisis, there was also no significant association between Beck’s Anxiety Inventory scores and the left uterine artery resistance index, (P=0.159, β=-0.227, 95% CI:-0.010 to 0.002) and right uterine artery resistance index (P=0.599, β=0.087, 95% CI:-0.023 to 0.039).

There was no significant relationship between Beck’s Anxiety Inventory score and change in the uterine artery resistance indexes.

**Table 2:** Association between uterine artery resistance index and Beck’s Anxiety Inventory scores


Variables	P value	Beta	95% confidence interval	
			Lower bound	Upper bound

Before amniocentesis				
Left uterine artery resistance index	0.394	0.141	-0.003	0.008
Right uterine artery resistance index	0.897	0.021	-0.005	0.006
Two weeks after amniocentesis				
Left uterine artery resistance index	0.159	-0.227	-0.010	0.002
Right uterine artery resistance index	0.599	0.087	-0.023	0.039


Dependent variable in each model: uterine artery resistance.

We did not observe any association between the State-Trait Anxiety Inventory scores and blood flow of the uterine arteries, before amniocentesis. However, 2 weeks after amniocentesis, there was a significant association between the State Anxiety Inventory score and the uterine artery resistance index (P =0.041, β=3.05, 95% CI:-2.548 to 8.072) and for the Trait Anxiety Inventory score (P=0.010, β=-23.82, 95% CI:-25.301 to-10.402). A significant relationship was observed between the State Anxiety Inventory scores and a decrease in the right uterine artery resistance index 2 weeks after amniocentesis (P=0.036, β=-0.274, 95% CI:-0.015 to-0.001, Tables3-5).

We found some associations between psychological scores and second trimester analytes. There was a significantly positive correlation between Beck’s Anxiety Inventory scores and the α-fetoprotein level (P<0.001, β=5.38). β-hCG levels were significantly higher in pregnant women with higher Trait Anxiety Inventory scores (P<0.001, β=3.94). Unconjugated estriol levels were significantly higher in pregnant women with higher State Anxiety Inventory scores (P=0.048, β=13.08).

**Table 3:** Associations between uterine artery resistance index and Trait Anxiety Inventory


Variables	P value	Beta	95% confidenceinterval	
			Lower bound	Upper bound

Before amniocentesis				
Left uterine arteryresistance index	0.940	-0.020	-0.011	0.10
Right uterine arteryresistance index	0.930	-0.023	-0.010	0.009
After amniocentesis				
Left uterine arteryresistance index	0.127	0.393	-0.002	0.019
Right uterine arteryresistance index	0.010	-23.82	-25.301	-10.402


Dependent variable in each model: uterine artery resistance.

**Table 4:** Associations between uterine artery resistance index and State Anxiety Inventory


Variables	P value	Beta	95% confidence interval for B	
			Lower bound	Upper bound

Before amniocentesis				
Left uterine artery resistance index	0.945	-0.018	-0.010	0.10
Right uterine artery resistance index	0.603	0.132	-0.035	0.059
After amniocentesis				
Left uterine artery resistance index	0.754	-0.078	-0.012	0.009
Right uterine artery resistance index	0.041	3.05	-2.548	8.072


Dependent variable in each model: uterine artery resistance.

**Table 5:** Associations between alterations of uterine artery resistance indexes before and 2 weeks after amniocentesis to Beck’s Anxiety Inventory and the State-Trait Anxiety Inventory


Dependent variables	P value	Beta	95% confidence interval for B	
			Lower bound	Upper bound

Beck’s Anxiety Inventory				
Left artery resistance index change	0.077	-0.287	-0.014	0.001
Right artery resistance index change	0.915	0.017	-0.007	0.008
Trait Anxiety Inventory				
Left artery resistance index change	0.197	0.332	-0.005	0.022
Right artery resistance index change	0.208	-0.310	-0.023	0.005
State Anxiety Inventory				
Left artery resistance index change	0.841	-0.201	-0.015	0.012
Right artery resistance index change	0.036	-0.274	-0.015	-0.001


Dependent variable in each model: uterine artery resistance.

## Discussion

During the antenatal period mothers face substantial anxiety, which may affect the prevalence of adverse obstetric outcomes. Some studies have found a correlation between psychological factors (depression, anxiety, and chronic stress) and preterm birth or low birth weight (4, 24, 25).

Since investigations have shown a higher prevalence of antenatal depression and anxiety disorders in pregnant Iranian women compared to the world average (26, 27), we assessed the role of psychological complications based on positive aneuploidy screening results in changes in uterine artery blood flow. Assessment of uterine artery blood flow by Doppler velocimetry is a valuable clinical tool to predict some adverse pregnancy outcomes (28, 29).

Studies have shown that informing pregnant women about the abnormal results of screening tests such as Down syndrome, or being aware of an invasive procedure like amniocentesis and waiting for its outcome causes tremendous distress and anxiety for these women.

The mean of the State-Trait Anxiety Inventory scores were more than 40 in our participants before amniocentesis, which showed a high anxiety level. In accordance with our results, Hoskovec and colleagues reported a significantly elevated mean state anxiety score in women with abnormal maternal serum screening tests. The score was significantly affected by factors like perceived risk and decision to undergo amniocentesis (30). Ng et al. (21), reported a mean trait anxiety score of 41.56 ± 6.35 in pregnant women before they underwent amniocentesis. In a prospective case-control study, Calışkan et al. (31) found that the maternal stress level based on Spielberger’s State-Trait Anxiety Inventory in 60 pregnant women who underwent amniocentesis (48.9 ± 11.8) was significantly higher than normal pregnant women (33.5 ± 6.5).

There was a significant relationship between the State-Trait Anxiety Inventory scores and a decrease in the right uterine artery resistance index 2 weeks after amniocentesis. This finding might show that a 2 week period could provide an opportunity for mothers to cope with their anxiety. Possibly, participants were relieved after receiving the negative amniocentesis results, which reduced their concerns about their foetus’ health status. In addition, high levels of anxiety could be associated with the amniocentesis process, which disappeared after 2 weeks. Possibly, the significant decrease in uterine artery resistance index could have been attributed to a decline in anxiety level.

Beck’s Anxiety Inventory score can be repeated after one week. We have administered the test after two weeks; however, we did not find significant changes despite the reduction in the State Anxiety score. The Trait Anxiety score is due to more chronic anxiety feelings, which we did not expect any changes in 2 weeks as was shown in our participants.

Several investigations have shown correlations between psychological scales and uterine artery blood flow indexes through alteration of the stress hormones corticotropin releasing hormone and cortisol (32, 33). Stress can also decrease the level of pregnancy supporting hormones like progesterone that increase prostaglandin production and result in increased uterine contractility, blood vessel resistance, and decreased uterine artery blood flow (25). Monk et al. (34) have reported that in 101 second trimester pregnant women who had a history of mental illness, there were no significant associations found between different measures of maternal depression (Beck Depression Inventory, Hamilton Rating Scale for Depression-17) or anxiety (Hamilton Rating Scale for Anxiety) and uterine artery blood flow indexes. Mendelson et al. (8) detected a linear association between decreased uterine artery resistance indexes and advancing gestational age during the second half of pregnancy; however, their cross-sectional analyses did not reveal any significant correlations between individual psychological scores and uterine artery resistance indexes.

The reason that we observed a relationship between scores and decline in the resistance index in the right uterine artery, but not the left, might relate to other factors like placental site. Although we did not consider the placental location in the present study, other investigations pointed to the effects of placental site on uterine artery flow and resistance (35, 36).

Our findings showed a significantly positive correlation between Beck’s Anxiety Inventory scores and α-fetoprotein levels. There was also a significant correlation between the unconjugated estriol levels and state scores. Altered α-fetoprotein and unconjugated estriol levels in depressed and anxious pregnant women might be due to alterations in the placental function and foetoplacental blood circulation (37-39).

Our results also showed a positive correlation between the trait anxiety scores and the second trimester β-hCG levels. On the other hand it seemed that pregnant women with high trait anxiety scores had abnormal patterns of uterine blood flow. Altered resistance index values, defective trophoblastic invasion, and transiently altered hormonal concentrations which might affect analyte transport might play a role in such abnormalities (31). Similar to our results, several studies have revealed interrelationships between high levels of β-hCG, nausea and vomiting during pregnancy, and psychological disturbances such as psychological stress (40). Oancea and colleagues found a significant correlation between high β-hCG levels in the second trimester and some maternal complications in 120 pregnant Caucasian women. They found that β-hCG had the highest predictive power for prenatal outcomes in the second trimester of pregnancy complicated by preeclampsia. This finding was also confirmed by Lee and Saha (40). To our knowledge, this study was among the few studies that focused on the association between psychological complications related to positive an euploidy screening serum analytes and changes in uterine artery blood flow. Such correlations could show the importance of stress management interventions to improve pregnancy outcome.

This study, like other pilot studies, had some methodological limitations. We did not consider other related factors like parity and gestational age, which have been shown to affect uterine artery resistance index in other studies (7). A larger sample size and well-designed study would be required to evaluate the differences.

## Conclusion

Maternal anxiety because of positive aneuploidy screening results and amniocentesis can affect perinatal outcomes via mood-based alterations in uterine artery blood flow resistance and screening markers like β-hCG, unconjugated estriol, and α-fetoprotein. We think that increasing pregnant women’s knowledge about the probability of false-positive results of screening tests, administration of proper psychological interventions, and psychological counselling and training may significantly decrease the influence of anxiety and improve pregnancy outcomes. Further studies are required on this subject with greater numbers of participants.

## References

[1] Bisetegn TA, Mihretie G, Muche T (2016). Prevalence and predictors of depression among pregnant women in Debretabor Town, Northwest Ethiopia. PLoS One.

[2] Loomans EM, van Dijk AE, Vrijkotte TG, van Eijsden M, Stronks K, Gemke RJ (2013). Psychosocial stress during pregnancy is related to adverse birth outcomes: results from a large multi-ethnic community-based birth cohort. Eur J Public Health.

[3] Non AL, Binder AM, Kubzansky LD, Michels KB (2014). Genome-wide DNA methylation in neonates exposed to maternal depression, anxiety, or SSRI medication during pregnancy. Epigenetics.

[4] Madhavanprabhakaran GK, D’Souza MS, Nairy KS (2015). Prevalence of pregnancy anxiety and associated factors. International Journal of Africa Nursing Sciences.

[5] Andersson L, Sundström-Poromaa I, Wulff M, Aström M, Bixo M (2004). Neonatal outcome following maternal antenatal depression and anxiety: a population-based study. Am J Epidemiol.

[6] Shirazi M, Azadi F, Shariat M, Niromanesh Sh, Shirazi M (2016). Effectiveness of stress management training on stress reduction in pregnant women. Tehran University Medical Journal.

[7] László KD, Liu XQ, Svensson T, Wikström AK, Li J, Olsen J (2013). Psychosocial stress related to the loss of a close relative the year before or during pregnancy and risk of preeclampsia. Hypertension.

[8] Mendelson T, DiPietro JA, Costigan KA, Chen P, Henderson JL (2011). Associations of maternal psychological factors with umbilical and uterine blood flow. J Psychosom Obstet Gynaecol.

[9] Vythilingum B, Geerts L, Fincham D, Roos A, Faure S, Jonkers J (2010). Association between antenatal distress and uterine artery pulsatilityindex. Arch Womens Ment Health.

[10] Fu J, Yang R, Ma X, Xia H (2014). Association between maternal psychological status and fetal hemodynamic circulation in late pregnancy. Chin Med J (Engl).

[11] Mousavi S, Hantoushzadeh S, Sheikh M, Shariat M (2015). Maternal anxiety and the second-trimester prenatal screening: a prospective cohort study. Fetal Diagn Ther.

[12] Baer RJ, Flessel MC, Jelliffe-Pawlowski LL, Goldman S, Hudgins L, Hull AD (2015). Detection rates for aneuploidy by first-trimester and sequential screening. Obstet Gynecol.

[13] reathnach FM, Malone FD, Lambert-Messerlian G, Cuckle HS, Porter TF, Nyberg DA (2007). First- and second-trimester screening: detection of aneuploidies other than down syndrome. Obstet Gynecol.

[14] Patrick J, Challis J, Natale R, Richardson B (1979). Circadian rhythms in maternal plasma cortisol, estrone, estradiol, and estriol at 34 to 35 weeks’ gestation. Am J Obstet Gynecol.

[15] Chou JY, Mano T, Feldman M (1982). Inhibition of synthesis of alpha-fetoprotein by glucocorticoids in cultured hepatoma cells. J Cell Biol.

[16] Mylonas I, Jeschke U, Winkler L, Makovitzky J, Richter DU, Briese V (2003). Immunohistochemical expression of inhibin-alpha in human endometrium and the in vitro secretion of inhibin, estradiol and cortisol in cultured human endometrial glandular cells. Arch Gynecol Obstet.

[17] Gagnon A, Wilson RD (2008). Society of Obstetricians and Gynaecologists of Canada Genetics Committee. J Obstet Gynaecol Can.

[18] Brajenović-Milić B, Martinac Dorcić T, Kuljanić K, Petrović O (2010). Stress and anxiety in relation to amniocentesis: do women who perceive their partners to be more involved in pregnancy feel less stressed and anxious?. Croat Med J.

[19] Kukulu K, Buldukoglu K, Keser I, Keser I, Simşek M, Mendilcioğlu I (2006). Psychological effects of amniocentesis on women and their spouses: importance of the testing period and genetic counseling. J Psychosom Obstet Gynaecol.

[20] Kowalcek I, Muhloff A, Bachmann S, Gembruch M (2002). Depressive reactions and stress related to prenatal medicine procedures. Ultrasound Obstet Gynecol.

[21] Ng CCM, Lai F M, Yeo GS (2004). Assessment of maternal anxiety levels before and after amniocentesis. Singapore Med J.

[22] Mahram B (1994). Standardization of Spielberger’s test anxiety inventory in Mashhad.Presented for M.Sc., Tehran.Allameh Tabatabaei University.

[23] Beck AT, Epstein N, Brown G, Steer RA (1988). An inventory for measuring clinical anxiety: Psychometric properties. J Consult Clin Psychol.

[24] Choi SK, Park YG, Park IY, Ko HS, Shin JC (2014). Impact of antenatal depression on perinatal outcomes and postpartum depression in Korean women. J Res Med Sci.

[25] Allison SJ, Stafford J, Anumba DO (2011). The effect of stress and anxiety associated with maternal prenatal diagnosis on feto-maternal attachment. BMC Womens Health.

[26] Karamoozian M, Askarizadeh G, Sanati K, Aminizadeh M (2015). Stress management on pregnancy women. J Nov Appl Sci.

[27] Mangoli M, Ramezani T, Alizadeh SM (2003). Screening mental disorders in pregnancy. Iran J Psychiatry Clin Psychol.

[28] Giordano R, Cacciatore A, Romano M, La Rosa B, Fonti I, Vigna R (2010). Uterine artery Doppler flow studies in obstetric practice. J Prenat Med.

[29] Barati M, Shahbazian N, Ahmadi L, Masihi S (2014). Diagnostic evaluation of uterine artery Doppler sonography for the prediction of adverse pregnancy outcomes. J Res Med Sci.

[30] Hoskovec J, Mastrobattista JM, Johnston D, Kerrigan A, Robbins-Furman P, Wicklund CA (2008). Anxiety and prenatal testing: do women with soft ultrasound findings have increased anxiety compared to women with other indications for testing?. Prenat Diagn.

[31] Calışkan E, Ozkan S, Cakıroğlu Y, Yalçınkaya O, Polat A, Corakçı A (2009). The effects of maternal anxiety prior to amniocentesis on uterine and fetal umbilical blood flow. J Turk Ger Gynecol Assoc.

[32] Florio P, Severi FM, Fiore G, Micheli L, Bocchi C, Nencini C (2003). Impaired uterine artery blood flow at mid gestation and low levels of maternal plasma corticotropin-releasing factor. J Soc Gynecol Investig.

[33] Bale TL, Baram TZ, Brown AS, Goldstein JM, Insel TR, McCarthy MM (2010). Early life programming and neurodevelopmental disorders. Biol Psychiatry.

[34] Monk C, Newport DJ, Korotkin JH, Long Q, Knight B, Stowe ZN (2012). Uterine blood flow in a psychiatric population: impact of maternal depression, anxiety, and psychotropic medication. Biol Psychiatry.

[35] Gupta V, Agarwal N (2014). Correlation between placental location and uterine artery flow waveforms in uncomplicated pregnancies. JIMSA.

[36] Oloyede OA, Iketubosin F (2013). Uterine artery Doppler study in second trimester of pregnancy. Pan Afr Med J.

[37] O’Donnell KJ, Bugge Jensen A, Freeman L, Khalife N, O’Connor T, Glover V (2012). Maternal prenatal anxiety and downregulation of placental 11β-HSD2. Psychoneuroendocrinology.

[38] Kane HS, Dunkel Schetter C, Glynn LM, Hobel CJ, Sandman CA (2014). Pregnancy anxiety and prenatal cortisol trajectories. Biol Psychol.

[39] Gitlin D, Boesman M (1967). 64 serum α-fetoprotein synthesis in the human and rat fetus and its inhibition in the rat. Pediatr Res.

[40] Lee NM, Saha S (2011). Nausea and vomiting of pregnancy. Gastroenterol Clin North Am.

